# Vaginal dilator use more than 9 months is a main prognostic factor for reducing G2‑late vaginal complications in 3D‑vaginal‑cuff brachytherapy (interventional radiotherapy)?

**DOI:** 10.1007/s12094-023-03099-4

**Published:** 2023-02-08

**Authors:** Yaowen Zhang, Faegheh Noorian, Rosa Abellana, José Rochera, Antonio Herreros, Gabriela Antelo, Valentina Lancellotta, Luca Tagliaferri, Qian Han, Aureli Torne, Angeles Rovirosa

**Affiliations:** 1 Cancer Center, Henan Provincial People’s Hospital, Zhengzhou, China; 2 Radiation Oncology Department, Hospital Clínic-Universitat de Barcelona, Barcelona, Spain; 3 Fonaments Clínics Department Faculty of Medicine, Universitat de Barcelona, C/ Casanova 153, 08036 Barcelona, Spain; 4 Dipartimento di Diagnostica per Immagini, U.O.C. Radioterapia Oncologica, Fondazione Policlinico Universitario A. Gemelli IRCCS, Radioterapia Oncologica ed Ematologia, 00168 Rome, Italy; 5 Gynecological Cancer Unit, Hospital Clinic-Universitat de Barcelona, Barcelona, Spain

**Keywords:** Late toxicity, Postoperative vaginal brachytherapy, Endometrial cancer, Vaginal dilators

## Abstract

**Purpose:**

Analyse the impact of different prognostic factors on G2-late vaginal complications after vaginal brachytherapy (VBT) ± external beam radiotherapy (EBRT) in postoperative endometrial cancer (PEC).

**Methods:**

One hundred and twenty-six PEC patients treated with VBT ± EBRT were retrospectively analysed considering age, body mass index, applicator diameter, clinical target volume (CTV), use of dilators, chemotherapy and EQD2_(α/β=3)_ at the most exposed 2 cm^3^ of the CTV as prognostic factors for vaginal complications. Late vaginal complications were evaluated using objective LENT-SOMA criteria. Statistics: descriptive analysis, Chi-square, Fisher and Student tests were applied. Univariate and multivariate analyses were performed with the Baptista–Pike exact method and multiple logistic regression.

**Results:**

Mean age was 65 years (SD ± 10), and median follow-up was 66 months (8–104). 19/126 patients (15%) showed G2-late vaginal complications, and 107/126 (85%) G0–G1. Univariate analysis showed: CTV ≤ 9 cm^3^ (*p* = 0.036), use of dilators < 9 months (*p* = 0.015), and total ≥ 68 Gy EQD2 received by 2 cm^3^ of CTV (*p* = 0.039) were associated with G2-late vaginal toxicity. Multivariate analysis showed the use of dilators < 9 months as an independent prognostic factor for G2-late vaginal toxicity (*p* = 0.043, OR 8.59, CI 1.59–159.9).

**Conclusion:**

The use of dilators < 9 months in VBT ± EBRT for PEC was an independent prognostic factor for G2-late vaginal toxicity. The use of vaginal dilators ≥ 9 months requires further analysis in studies evaluating late vaginal toxicity.

## Introduction

Endometrial cancer (EC) is the most common gynaecological cancer in women in developed countries. Surgery is the main treatment and adjuvant radiotherapy is used according to pathologic and molecular prognostic factors described in many studies and consensus documents [1]. The use of vaginal brachytherapy (VBT) (or vaginal interventional radiotherapy) maximises local control and minimises adverse effects. Late vaginal complications (LVC) after VBT ± external beam radiotherapy (EBRT) for postoperative EC (PEC) treatment have been little analysed and are usually reported as G1–G2 and with an incidence of up to 50% depending on the series [2, 3]. G1-LVC has no clinical impact but G2-LVC manifests as vaginal bleeding, painful intercourse, and stenosis of the upper third of the vagina. The latter makes gynaecological exams, pap-smear practice and intercourse difficult and can cause physical and psychological problems [4].

Some studies on possible associated risk factors and prevention methods have had unclear results. The risk factors associated with LVC are age, stage, vaginal length treated, dose administered, body mass index (BMI), chemotherapy, applicator diameter, vaginal dilator (VD) use and treatment modality [5]. Nevertheless, there is no consensus among experts and organisations considering all the possible prognostic factors for G2-LVC and they are not usually analysed in a single study. Moreover, the number of patients included in the analyses is commonly low and some data may be missing in retrospective studies.

Three studies performed in our centre, in which patients were recruited prospectively and analysed retrospectively, showed a relationship between vaginal EQD2_(α/β=3)_ and G2-LVC [2, 6, 7]. However, other possible prognostic factors were not considered.

The present study was aimed at analysing the impact of the different prognostic factors on G2-LVC in PEC patients treated with VBT ± EBRT. This is the first study to analyse the possible prognostic factors for G2-LVC in patients with a similar clinical target volume (CTV) delineation and lengthy patient follow-up.

## Materials and methods

The present study was approved by the Institutional Ethical Review Board and informed consent was obtained from all the patients (HCB 2022/0379).

From December-2013 to December-2018, 126-PEC patients treated with VBT ± EBRT were prospectively included in the present analysis.

After endometrial biopsy and imaging studies, all the patients underwent surgery as the main treatment according to the recommendations of International Federation of Gynaecology and Obstetrics (FIGO) 2009 or the European Society for Medical Oncology 2016 guidelines. The surgical approaches included: vaginal hysterectomy plus bilateral salpingo-oophorectomy (HBO) and laparoscopic pelvic ± para-aortic lymphadenectomy 45/126 (35.7%); abdominal HBO + pelvic lymphadenectomy 11/126 (8.7%); the former plus para-aortic lymphadenectomy and omentectomy 9/126 (7.1%); exclusive HBO 52/126 (41.3%), simple hysterectomy 5/126 (4.0%) and other surgical procedures 4/126 (3.2%).

According to the pathological characteristics, our multidisciplinary Gynaecological Cancer Unit (GCU) classified 63/126 (50%) patients as in the intermediate-risk group (type-1 endometrial cancer, IA-G2, G3 or IB-G1G2, or type-2 IA G1-3) and the remaining 63/126 (50%) as the high-risk/advanced group (intermediate risk with vascular and lymphatic space invasion [VLSI] or IB-G3 and II–IVA stages).

Table 1 shows the pathological characteristics of the 126 patients. In the intermediate-risk group, exclusive VBT was administered using two different schedules**: **6Gyx3 fractions (EQD2_(α/β=10)_ = 27 Gy) (31/126) or 7.5 Gy × 2-fractions (32/126) (EQD2_(α/β=10)_ = 21.88 Gy). Combined treatment was administered in the high-risk/advanced group. A median dose of 45 Gy (45–50.4), 1.8-2 Gy/fraction in 5-fractions/week was given followed by one 7 Gy fraction of VBT (EQD2_(α/β=10)_ ranged between = 54.2 Gy and 59.9 Gy depending on the EBRT dose). Chemotherapy was administered before radiotherapy in 23/63 (36.5%) high-risk stage I and II–IVA stage patients according to age and comorbidities (4–6 courses cisplatin + carbo taxol/3 weeks).

**Table 1:** Pathological characteristics of the patients

	Intermediate-risk group (63 p)	High-risk group (63 p)	Total
Mean age ± SD (years)	65.0 ± 9.32	64.0 ± 10.69	64.5 ± 9.99
Median age and range	67 (41–86)	65 (40–84)	66 (40–86)
2009 FIGO stage:			
IA	44 (69.8%)	16 (25.4%)	60(47.6%)
IB	17 (27.0%)	19 (30.2%)	36(28.6%)
II	2 (3.2%)	8 (12.7%)	10(7.9%)
IIIA	0 (0%)	2 (3.2%)	2 (1.6%)
IIIB	0 (0%)	1 (1.6%)	1 (0.8%)
IIIC1	0 (0%)	6 (9.5%)	6 (4.8%)
IIIC2	0 (0%)	6 (9.5%)	6 (4.8%)
IVA	0 (0%)	2 (3.2%)	2 (1.6%)
IVB	0 (0%)	3 (4.8%)	3 (2.4%)
Grade			
1	22 (34.9%)	7 (11.1%)	29 (23.0%)
2	32 (50.8%)	35 (55.6%)	67 (53.2%)
3	9 (14.3%)	21 (33.3%)	30 (23.8%)
Pathological type:			
Endometrioid	58 (92.1%)	51 (81.0%)	109 (86.5%)
Serous	0 (0%)	7 (11.1%)	7 (5.5%)
Clear cells	2 (3.2%)	3 (4.8%)	5 (4.0%)
Mixed	3 (4.8%)	2 (3.2%)	5 (4.0%)
Myometrial invasion			
≤ 50%	46 (73.0%)	27 (42.9%)	73 (58.0%)
> 50%	17 (27.0%)	36 (57.1%)	53 (42.0%)
VLSI			
No	56 (88.8%)	41 (65.1%)	97 (77.0%)
Yes	5 (8.0%)	19 (30.1%)	24 (19.0%)
Missing	2 (3.2%)	3 (4.8%)	5 (4.0%)
Mean tumour size (cm) and SD	2.9 ± 1.32	3.6 ± 1.76	3.3 ± 1.58
Median tumour size and range	2.7 (0.5–7)	3.5 (1–10)	3 (0.5–10)

*SD* Standard deviation, *VLSI* vascular and lymphatic space invasion

### External beam irradiation

After obtaining images from a specific computerised tomography (CT) scan, CTV delineation was performed following the Radiation Therapy Oncology Group guidelines [8]. In 60 patients, radiotherapy was 3D-planned and administered in the pelvic ± para-aortic region using an isocentric four-field technique and 6/18 MV photon beams. Intensity-modulated radiotherapy (6 MV) was administered in three patients. All patients were treated five times/week.

### Vaginal-cuff brachytherapy

Brachytherapy was administered at 1–2 weeks after completing EBRT. The first applicator placement was performed in the operating room, where patients were examined to confirm correct healing of the vaginal wound and determine the type and diameter of applicator required. The vaginal cylinder technique was used in 121/126 (96%) patients, with a cylinder diameter of 2.5 cm in 4 (3.3%) patients, 3 cm in 18 (14.9%) patients and 3.5 cm in 99 (81.8%) patients; colpostats were used in the 5 (4%) remaining patients. CT images were obtained with a slice thickness of 1 mm for 3D-planning. After defining the CTV and organs at risk (OAR), treatment planning was performed in each patient. The CTV was delineated along the first cylinder with automatic 3 mm expansion from the cylinder and then the exclusion of the cylinder based on Hounsfield number discrimination was performed. Thereafter, the CTV was manually corrected for excess or defect of the thickness of the vaginal wall obtained after automatic generation**.** OARs in (bladder, sigmoid, small bowel and rectum) were delimited outlining the outer contour. The CTV of the vagina was duplicated and also considered as an OAR. The CTV of the vagina is considered the same as the OAR for vagina. The technique and procedure are described elsewhere [6]. The dose was prescribed at 5 mm from the applicator surface with optimisation of dose distribution. The active source treatment length was 2.5 cm in the cylinder cases and was adapted to the needs of each patient in the case of colpostats. The Oncentra Brachy planning system (V.4.5.3) (Elekta^®^, Nucletron BV, Veenendaal, The Netherlands) was the software used for treatment planning.

For the analysis of LVT, the dose equivalent to 2 Gy/fraction (EQD2) considering α/β = 3 was calculated for each patient for exclusive VBT and VBT + EBRT (sum of both doses) at 2 cm^3^ of the most exposed part of the dose in the CTV. This 2 cm^3^ of most exposed dose are located at the top of the treated vaginal cuff.

The patients were followed at 15 days after the end of treatment, every 3–4 months in the first 2 years and every 6 months thereafter until 5 years. Patients were strongly encouraged to use VD daily if possible. The presence of local, pelvic, or distant recurrence was assessed by clinical and radiological methods according to the GCU protocol. Intercourse activity was not recorded. The presence of VLC was determined by gynaecological examination, clinical interview and, when necessary, radiological tests, among others. Rectal, bladder and small bowel late toxicity were graded using RTOG criteria, and the objective criteria of LENT-SOMA were used for grading LVC and scored prospectively: G1 atrophy, telangiectasia adherences, < 1/3 shortened vaginal length; G2 bleeding telangiectasias, symptomatic dryness, 1/3 and 2/3 shortened vaginal length, partial synechiae [9, 10].

The following prognostic factors were considered for G2-LVC in the univariate/multivariate analyses: age ≤ 55 years vs. > 55 years, BMI ≤ 35 vs. > 35, applicator diameter < 3.5 cm vs. 3.5 cm, CTV ≤ 9 cm^3^ vs. > 9 cm^3^, non-use of VD or use < 9 months vs. ≥ 9 months, chemotherapy yes or no, and EQD2_(α/β=3 Gy)_ at 2 cm^3^ of the most exposed volume of dose in the CTV < 68 Gy vs. ≥ 68 Gy.

### Statistical analysis

Categorical data are expressed using frequencies and percentages, while continuous data are expressed using means and standard deviations. A Chi-square-test was used to study the association between the outcome and categorical variables and the Student’s-test in case of continuous variables. The 95% confidence interval (95% CI) for the odds ratios (OR) was computed using the Baptista–Pike exact method. All the variables associated with G2-LVC (age, BMI, applicator diameter, CTV, use of VD, chemotherapy and EQD2_(α/β=3 Gy)_ at the most exposed 2 cm^3^ of the CTV) in the univariate analysis were considered in the multiple logistic regression. The type I error was set at 5%. The analyses were performed using R-software for Windows version 4.0.3 (R project for statistical computing; Vienna, Austria).

## Results

The mean age of the patients was 65 years (SD ± 10) and the median follow-up was 66 months (8–104). No patient presented vaginal-cuff relapse. Table 2 describes the different prognostic factors and their association with G2-LVC. In most of the patients using VD, the adherence was ≥ 2–3 times/week. In the present series, 19/126 (15%) patients presented G2-LVC, while 107/126 (85%) patients had G0–G1. In 62/126 (49.2%) patients, LVC was considered as G0. In 45/126 (35.7%) patients, G1-LVC was manifested as telangiectasia in vaginal mucosa (26/45 (57.7%)), as “small dog ear” (16/45 (35.5%)) or small adhesions that could be reopened during clinical examination (3/45 (6.6%)). G2-LVC appeared as vaginal shortening > 1/3(13/19, (68.4%)) or contact bleeding mainly by the presence of telangiectasia (6/19; (31.6%)). The median time to the appearance of G1-LVC was 22 months (7–49) and 11 months (8–51) for G2-LVC, with the latter appearing in 9/19 patients after 11 months. The dose received to the 2 cm^3^ dose volume was different considering the exclusive VBT vs. EBRT + VBT treatments: in exclusive VBT the mean dose was 62.3 Gy ± 9.0 and the median dose was 61 Gy with a range between 49.2 Gy and 113 Gy. While in EBRT + VBT, the mean dose was 73 ± 8.6 and the median was 70.1 Gy with a range between 55.5 Gy and 107.9 Gy.

**Table 2:** Prognostic factors for ≥ G2-late vaginal complications in the univariate analysis

	G0–G1: 107	G2: 19	OR (95% CI)	*P* value	Number
Mean age ± SD (years)	64.0 ± 10.1	67.2 ± 8.93	1.03 (0.98; 1.09)		
≤ 55	23 (95.8%)	1 (4.17%)	–	0.177	126
> 55	84 (82.4%)	18 (17.6%)	4.34 (0.82; 108)	0.121	126
BMI				1.000	
< 35	54 (84.4%)	10 (15.6%)	–		83
≥ 35	16 (84.2%)	3 (15.8%)	1.04 (0.20; 4.00)		43
Missing	37	6			
Cylinder diameter				0.374	126
3.5	86 (86.9%)	13 (13.1%)	–		
< 3.5	17 (77.3%)	5 (22.7%)	1.96 (0.55; 6.08)		
Colpostat	4 (80.0%)	1 (20.0%)	1.80 (0.06; 14.3)		
CTV				0.036	125
> 9 cc	36 (94.7%)	2 (5.26%)	–		
≤ 9 cc	70 (80.5%)	17 (19.5%	4.08 (1.07; 29.1)		
Missing	1	0			1
Vaginal dilator use				0.015	115
≥ 9 months	39 (97.5%)	1 (2.50%)	–		
< 9 months	59 (78.7%)	16 (21.3%)	9.25 (1.76; 229)		
Missing	9	2			11
Chemotherapy				0.319	112
No	77 (88.5%)	10 (11.5%)	–		
Yes	20 (80.0%)	5 (20.0%)	1.93 (0.54; 6.23)		
Missing	10	4			14
EQD2				0.039	126
< 68 Gy	59 (92.2%)	5 (7.81%)	–		
≥ 68 Gy	48 (77.4%)	14 (22.6%)	3.35 (1.17; 11.2)		
EQD2 in dilators < 9 months				0.273	75
< 68 Gy	28 (84.8%)	5 (15.2%)	–		32
≥ 68 Gy	31 (73.8%)	11 (26.2%)	1.98 (0.61; 6.57)		42
EQD2 in dilators ≥ 9 months				0.106	40
< 68 Gy	27 (100%)	0 (0.00%)	–		27
≥ 68 Gy	12 (92.3%)	1 (7.69%)	6.6 (0.05; 82.80)		13

*BMI* body mass index, *CTV* clinical target volume, *EQD2* equivalent dose in 2 Gy/fraction, *SD* standard deviation

### Univariate analysis

Table 2 shows the results of the univariate analysis. G2-LVC was presented by 19.5% of the women with a CTV ≤ 9 cm^3^ compared with 5.3% with CTV > 9 cm^3^ (*p* = 0.036). In addition, VD use and the total EQD2 received by the 2 cm^3^ most exposed CTV volume was associated with G2-LVC. Women using VD ≥ 9 months presented 2.5% of G2-LVC while 21.3% of those using VD < 9 months presented this complication (*p* = 0.015). Regarding the total EQD2 received by 2 cm^3^ of the CTV, the proportion of G2-LVC was higher for doses ≥ 68 Gy (26.2% vs. 12.5%) (*p* = 0.039). Moreover, among the patients with a smaller CTV developing G2-LVC, all but one received EQD2 to 2 cm^3^ of the CTV > 68 Gy.

### Multivariate analysis

Table 3 shows the results of the multivariate logistic regression analysis. Of the three factors associated with G2-LVC in the univariate analysis, multivariate analysis identified VD use as an independent prognostic factor for G2-LVC (OR = 8.59; 95% CI 1.59; 159.9).

**Table 3:** Three prognostic factors for G2 vaginal toxicity in the multivariate analysis

Variables	Estimate	SE	*P* value	OR (95% CI)
CTV ≤ 9 cc	1.058	0.087	0.190	2.88 (0.70, 19.56)
Dilators < 9 months	2.151	1.063	0.043	8.59 (1.59, 159.92)
EQD2 ≥ 68 Gy	0.713	0.598	0.233	2.04 (0.66, 7.12)

*SE* standard error,* OR* odds ratio,* CTV* clinical tumour volume,* EQD2* equivalent dose in 2 Gy/fraction

## Discussion

VBT ± EBRT is a well-known treatment in PEC patients. Due to the good survival outcomes and low toxicity, few studies have specifically evaluated the effects of G2-LVC. The estimated incidence of different degrees of LVC after radiotherapy can be as high as 50% depending on the series [11]. The sexual and psychosexual problems of LVC have been reported in 40–100% of gynaecological cancer survivors, reducing their quality of life [12].

The present study highlights the different prognostic factors for G2-LVC in PEC patients treated with 3D-VBT ± EBRT. The results showed that VD use ≥ 9 months is an independent prognostic factor for decreasing G2-LVC.

Age has also been considered to be associated with radiation-induced vaginal stenosis. Previous studies have reported that age > 50 years is a significant predictive factor of stenosis, which can be explained by the lack of oestrogen and/or sexual activity in older women, leading to thinning, dryness and atrophy of the vaginal mucosa making the appearance of mucositis and fibrosis after radiotherapy more likely [13]. In contrast, similar to our results, Hartman and Diddle found no correlation between age and vaginal stenosis, making further studies necessary to draw consistent conclusions [14]. Regarding the BMI, several studies have assessed its effect on doses to OARs in VBT. A significant inverse correlation was found between BMI and doses to the sigmoid colon and bladder; the lower the BMI, the higher the doses to the OARs [15, 16]. Therefore, it could be hypothesised that a lower BMI is related to the doses received to a wider vaginal wall, and thus, to greater vaginal toxicity. In the present series, the role of BMI in vaginal toxicity was evaluated and showed no relationship between the two.

Applicator diameter is linked with LVC. Patients with small cylinders < 2.5 cm presented a higher incidence of G1-2 vaginal stenosis [17]. The present series found no significant association between vaginal cylinders ≤ 3 cm and chemotherapy and G2-LVC (the number of 2.5 cm cylinders was low). A study by Nieto et al*.* compared the adverse effects of EC patients receiving BT with or without chemotherapy and showed that the rate of vaginal stenosis did not rise with the addition of chemotherapy [18].

The irradiated vaginal volume has also been reported as a significant risk factor for LVC in several observational studies of VBT-treated EC patients [11, 19, 20]. A multivariable analysis by Henry Park et al*.* in 101 patients reported that > 60% of treated vagina was an independent predictor of ≥ G1 vaginal stenosis [20]. High vaginal toxicity may have also been due to the irradiation of the lower third of the vagina, which was sensitive to radiotherapy [21]. In the present series, the treated vagina length was 3 cm and in the univariate analysis, a CTV ≤ 9 cm^3^ was associated with G2-LVC (*p* = 0.036). The latter can probably be explained by the fact that among 87 patients with a CTV ≤ 9 cm^3^, 17 cases, most of whom (13/17(76%)) had received an EQD2 ≥ 68 Gy, presented complications. In contrast, in the group without G2-LVC, about half (33/70, [47%]) received a high dose (≥ 68 Gy). Therefore, we can hypothesise that higher doses in cases of CTV ≤ 9 cm^3^ increase the possibility of G2 vaginal complications, and therefore, the dose prescription to the CTV instead of at 5 mm from the applicator surface would resolve this increased incidence of G2-LVC.

Similar to our previous results, the EQD2_(α/β=3)_ ≥ 68 Gy at 2 cm^3^ of the CTV increased the risk of G2-LVC in the present series [2, 6, 7]. Susko et al*.* reported a significant cut-off of 130 Gy EQD2_(α/β=3 Gy)_ at 2 cm^3^ for ≥ G2-LVC [22]. A relationship between vaginal dose and LVC has been reported previously [23].

The univariate analysis in the present study also showed that VD use ≥ 9 months was useful for decreasing the incidence of vaginal shortening. The median time to G2-LVC was 11 months, thereby suggesting that patients would benefit from VD use for > 9 months. In the early response phase, VD can separate early adhesions and prevent posterior vaginal wall closure. They can also stretch vaginal tissue and promote epithelial cell growth, inhibiting elastosis and the proliferation of fibrous tissue [24]. Moreover, regular use improves sexual function and may gradually return the vagina to normal size [25].

The effectiveness of VD in preventing vaginal stenosis has been analysed with inconclusive results. Varghese et al*.* showed that VD were effective for 60% of users compared to 20% of non-users (*p* = 0.007) at 9 months of follow-up [26]. In another study on VD use, 80% of the patients presented no vaginal stenosis, 10% partial stenosis and 2.2% radiation necrosis at 12 months after radiotherapy. The authors reported that adhering to VD use can effectively reduce the grade of vaginal stenosis [27]. Bahng et al*.* reported a significant correlation between VD use at least 2–3 times/week and a lower risk of LVC on multivariate analysis [11]. Similarly, another multivariate analysis of 243 stage I–II patients with a median follow-up of 15 months analysed various factors such as age, BMI, ethnicity, gravidity, sexual activity, months of cylinder use, vaginal length, histological FIGO grade, histology, radiation dose-fractionation, receipt of chemotherapy, vaginal percent length treated, VD compliance, extended compliance, and frequency of dilator use. The authors found that the lack of consistent VD use (≥ 2 times/week for a minimum of 1 year) was an independent predictive factor of ≥ G1 vaginal stenosis (*p* < 0.05)[28]. Vagal et al*.* described similar results in patients receiving VD therapy [29]. Despite the lack of more multivariate analyses and the last Cochrane report on VD use being inconclusive, international guidelines recommend their use to reduce vaginal stenosis [4, 30]. Nevertheless, the results of our multivariate analysis show that improving compliance with VD is helpful for preventing vaginal stenosis in PEC treated with VBT ± EBRT. Our patients were encouraged to use VD during the 5-year follow-up. Multivariate analysis showed that VD use for < 9 months was an independent prognostic factor for vaginal shortening. Indeed, the median time to G2-LVC was 11 months. Previous series analysing VD use were performed during a maximum period of one year with inconclusive results in some. Considering that G2-LVC appeared later during follow-up, VD use for ≥ 9 months makes sense.

We also compared EQD2_(α/β=3 Gy)_ at 2 cm^3^ of the vagina with < 68 Gy and ≥ 68 Gy in patients using VD < 9 months and ≥ 9 months, respectively. Among patients using VD < 9 months, 11/16 (68.8%) women with G2-LVC received doses ≥ 68 Gy, and 31/59 (52.5%) women without G2-LVC received higher doses. No significant difference was found between the two dose groups. Therefore, patients using VD less time presented G2 toxicity likely not due to the doses received. Only one patient using VD ≥ 9 months and receiving EQD2_(α/β=3 Gy)_ ≥ 68 Gy developed G2-LVC. The dose administered may have influenced the appearance of complications similar to what previous studies by our group and other authors have described. Nevertheless, in the present study, the question regarding a possible relationship between the dose and VD use remains to be analysed and might be resolved in the prospective study ongoing in our centre.

One of the limitations of this study is the sample size and also that data on sexual activity were not collected. Moreover, the incidence of G2-LVC was low, leading to the need for the inclusion of more patients in a prospective analysis to determine possible differences. Nevertheless, this is the first study with a multivariate analysis showing the benefits of VD use for > 9 months in patients with a similar CTV definition, active source treatment length, 3D-planning, and a lengthy follow-up.

## Conclusions

Although in the univariate analysis a CTV < 9 cm^3^, VD use < 9 months and vaginal D2cc EQD2_(α/β=3)_ ≥ 68 Gy were found to be prognostic factors for G2-LVC, in the multivariate analysis, VD use ≥ 9 months was an independent prognostic factor for the prevention of G2-LVC. As this is the first study showing this finding in patients with homogeneous 3D-planning treatment and a lengthy follow-up, VD use ≥ 9 months should be analysed more in-depth in the future and included in all studies analysing LVC.
